# FE-YOLO: An Efficient Deep Learning Model Based on Feature-Enhanced YOLOv7 for Microalgae Identification and Detection

**DOI:** 10.3390/biomimetics10010062

**Published:** 2025-01-16

**Authors:** Gege Ding, Yuhang Shi, Zhenquan Liu, Yanjuan Wang, Zhixuan Yao, Dan Zhou, Xuexiu Zhu, Yiqin Li

**Affiliations:** 1 China Waterborne Transport Research Institute, Beijing 100088, China; dgglock@outlook.com (G.D.); yaozhixuan@wti.ac (Z.Y.); zhoudan@wti.ac.cn (D.Z.); zhuxuexiu@wti.ac.cn (X.Z.); 2 School of Railway Intelligent Engineering, Dalian Jiaotong University, Dalian 116028, China; hang9979@163.com (Y.S.); liuzhenquan1633@163.com (Z.L.); wangyanjuan@djtu.edu.cn (Y.W.)

**Keywords:** microalgal detection, feature fusion, object detection, deep learning

## Abstract

The identification and detection of microalgae are essential for the development and utilization of microalgae resources. Traditional methods for microalgae identification and detection have many limitations. Herein, a Feature-Enhanced YOLOv7 (FE-YOLO) model for microalgae cell identification and detection is proposed. Firstly, the feature extraction capability was enhanced by integrating the CAGS (Coordinate Attention Group Shuffle Convolution) attention module into the Neck section. Secondly, the SIoU (SCYLLA-IoU) algorithm was employed to replace the CIoU (Complete IoU) loss function in the original model, addressing the issues of unstable convergence. Finally, we captured and constructed a microalgae dataset containing 6300 images of seven species of microalgae, addressing the issue of a lack of microalgae cell datasets. Compared to the YOLOv7 model, the proposed method shows greatly improved average Precision, Recall, mAP@50, and mAP@95; our proposed algorithm achieved increases of 9.6%, 1.9%, 9.7%, and 6.9%, respectively. In addition, the average detection time of a single image was 0.0455 s, marking a 9.2% improvement.

## 1. Introduction

Microalgae are widely distributed on Earth and applied in various fields such as biomedicine, new energy, food, and healthcare, with over 20,000 known species. Before their development and utilization, accurate species identification is essential. The detection and identification of microalgae are crucial for unlocking their full potential in biofuel production, pharmaceutical development, and environmental protection. Moreover, microalgae have inspired innovations in biomimicry, with their efficient photosynthesis mechanisms serving as models for advanced solar energy systems, while their unique structural properties have led to biomimetic materials used in sensors, coatings, and drug delivery systems [1]. For example, F. Zhang et al. [2] explored the use of microalgae in biohybrid microrobots, applying biomimicry by drawing inspiration from the propulsion and phototaxis behaviors of natural microalgae. The study also discusses methods for functionalizing the microalgae surface to enhance their performance, with potential applications in drug delivery, imaging, and water purification. Finally, the authors highlight the future potential and challenges of this biomimetic technology. Thus, microalgae represent a promising resource not only for practical applications in multiple industries but also for advancing biomimetic technologies, unlocking new possibilities for sustainable solutions and innovative designs.

Since its introduction in 2006, deep learning technology has become the most discussed research direction in artificial intelligence and is quickly becoming the focal point of global research [3]. The introduction of deep learning has brought new approaches to the identification and detection of microalgae. In algae research, Qian et al. [4] proposed a novel multi-object deep learning framework for algae analysis based on Faster R-CNN. This framework can simultaneously address various tasks such as genus classification, algae detection, and organism identification. Although this work achieved multi-objective detection, it still faces the challenge of insufficient accuracy. Samantaray A et al. [5] proposed a computer vision system based on deep learning for algae monitoring with a wide range of applicable platforms but only 82% accuracy. Cho S. et al. [6] and Deglint J. L. et al. [7] explored the potential application of deep learning on algae in conjunction with 3D printing and other devices, achieving sufficient accuracy, but at a high cost. Wang et al. [8] introduced an improved Faster R-CNN model using Residual Network 50 (ResNet-50) and the Feature Pyramid Network (FPN) module to enhance feature extraction and address multi-scale target detection, effectively reducing missed detections. To enhance identification accuracy, Cao [9] proposed a ballast water microalgae identification method based on an improved YOLOv3 model. This method employs a lightweight MobileNet network instead of the original Darknet-53 and introduces an enhanced spatial pyramid pool (SPP) while optimizing the YOLOv3 loss function with the Complete IoU (CIoU) algorithm. Although these improvements increased detection accuracy, issues of missed detections remained. Pant [10] enhanced the original ResNeXt CNN model by reducing the size of convolutional kernels and filters, achieving high accuracy in differentiating discoidal algal genera. Yet, the model’s generalization was limited due to the homogeneous nature of the dataset’s algal species. Krause et al. [11] used a fully convolutional neural network to predict bounding boxes for detecting diatoms in microscope images, achieving good results in accuracy, speed, and reduced leakage detection. However, the small dataset influenced detection outcomes. Jianhong Dong et al. [12] studied the M-YOLO v8s model by replacing the C2F module and introducing Focal SIOU loss, optimizing the network structure, improving accuracy (accuracy increased to 98.9%) and detection speed, while reducing computational resource consumption (parameters and FLOPs were significantly reduced). However, the model may exhibit instability under certain extreme microalgae image conditions, and further optimization of image preprocessing methods is needed to improve adaptability.

Given that the YOLOv7 algorithm demonstrates efficient real-time object detection capabilities, enabling the rapid identification of small microalgae cells while maintaining high accuracy, it is particularly well-suited for handling complex backgrounds and densely packed targets, making it an ideal choice for microalgae detection. Therefore, we propose improvements to the YOLOv7 algorithm to further enhance its performance in this specific application [13]. To address the existing constraints of microalgae detection methods, a novel approach for microalgae identification and detection is proposed, based on global information and feature fusion. Firstly, we propose adding CAGS to our network structure. CAGS processes the feature maps by considering both width and height, utilizing depth-wise separable convolution (DWConv) [14] and channel shuffle mechanisms [15]. This enhancement boosts the network’s feature extraction capability without increasing the computational load. In this paper, we adopt SIoU [14] as the loss function for our method, addressing the issue of unstable network convergence that arises when the aspect ratio of the predicted frame matches that of the real frame. Furthermore, we acquired seven common microalgal samples, including *Chaetoceros*, *Chlorella*, *Chrysophyta*, *Prorocentrum lima*, *Karenia*, *Dunaliella*, and *Phaeodactylum*. A dataset containing 6300 images was created using microscopic photography. The proposed method was tested and compared with the latest classical algorithms using this dataset. The experiments indicate that the FE-YOLO proposed in this study shows significant improvements over other state-of-the-art methods in terms of accuracy, Recall, mAP@0.5, mAP@0.95, and other metrics.

The research conducted in this paper is of significant importance for the rapid identification and detection of microalgal cells, the advancement and utilization of microalgal resources, the protection of marine ecological environments, and the mitigation of harmful algal bloom disasters. This study provides a crucial foundation for enhancing the efficiency and accuracy of microalgal detection methods, which can lead to better resource management and environmental protection strategies. Furthermore, the findings of this research align with the growing field of biomimicry, as microalgae’s natural processes serve as a model for developing more efficient detection systems. By mimicking biological mechanisms, such as the way organisms recognize and respond to environmental cues, these advanced detection methods can improve not only the speed and accuracy of microalgal identification but also their application in sustainable technologies [16]. This research, therefore, plays a key role in advancing both the utilization of microalgae and the protection of marine ecosystems, supporting the development of smarter, more adaptive systems for monitoring water quality and mitigating environmental hazards.

## 2. Materials and Methods

### 2.1. Dataset Construction and Processing

The dataset forms the foundation of object detection, with the quality of the data determining the upper limit of detection accuracy. Currently, one of the primary challenges in the field of microalgae detection is the lack of high-quality public datasets, which significantly hinders the development of intelligent microalgae detection technologies.

**Figure 1:**
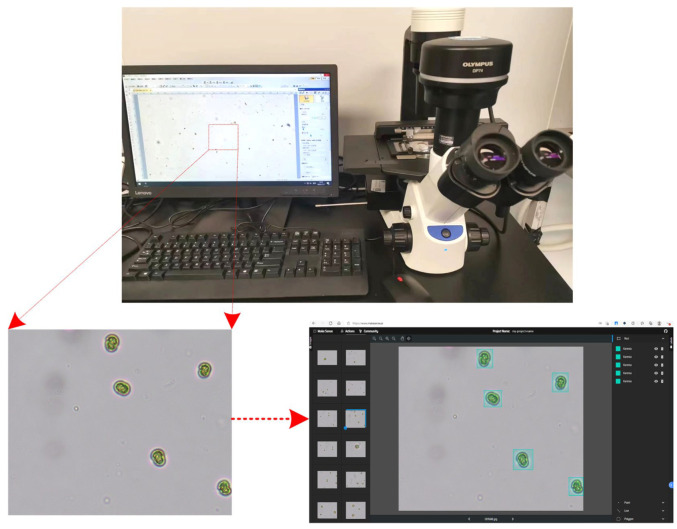
Microalgae image acquisition device.

In this study, seven common microalgae species—*Chaetoceros*, *Chlorella*, *Chrysophyta*, *Prorocentrum lima*, *Karenia*, *Dunaliella*, and *Phaeodactylum* (all sourced from the Liaoning Marine Technology Research Institute)—were selected as experimental samples. A microalgae dataset comprising images was established using microscopy (Olympus CKX53, Microscope Central, Feasterville, PA, USA) for image acquisition. A microscope was used to capture images under a 40× objective lens and the MakeSense online annotation tool was used for annotation, with the annotation files stored in YOLO format, as shown in Figure 1. Initially, 2100 microalgae images were captured using a microscope. To prevent overfitting due to insufficient data, data augmentation techniques, including the addition of salt-and-pepper noise and random scaling, were applied to the images. This process expanded the dataset to a total of 6300 images, ensuring an equal number of images for each algae species.

### 2.2. FE-YOLO Algorithm

YOLOv7 faces challenges in detecting small and densely packed microalgae cells in the dataset, as well as issues with background interference and instability in model convergence, necessitating algorithmic improvements for enhanced accuracy and robustness. To address these challenges, this paper proposes the FE-YOLO algorithm. First, the CAGS module is integrated into the Neck section of YOLOv7 to enhance detection accuracy without sacrificing training or inference speed. Second, the SIoU loss function is adopted in place of CIoU to improve model convergence stability and further enhance detection performance.

#### 2.2.1. Optimized Network Architecture

**Figure 2:**
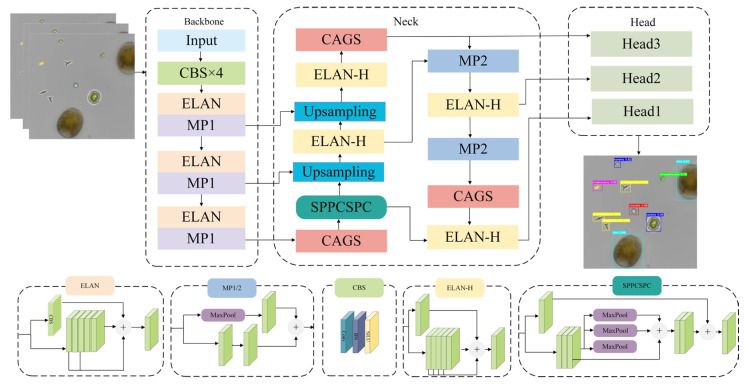
FE-YOLO model structure.

The FE-YOLO model structure proposed in this paper is illustrated in Figure 2. The CAGS module is incorporated into the Neck section, allowing features to be fused while considering both channel and directional correlations without adding additional computational overhead. By optimizing the network structure and introducing the CAGS module, we not only improved training and detection speeds but also enhanced feature extraction capabilities, leading to superior performance in microalgae detection tasks.

#### 2.2.2. CAGS Module

The Coordinate Attention (CA) mechanism [17] effectively understands and utilizes information from different channels and positions in input feature maps. However, this mechanism struggles to capture relationships between specific positions when handling long-range dependencies. As a result, the CA attention mechanism performs poorly in capturing correlations between spatially distant pixels.

**Figure 3:**
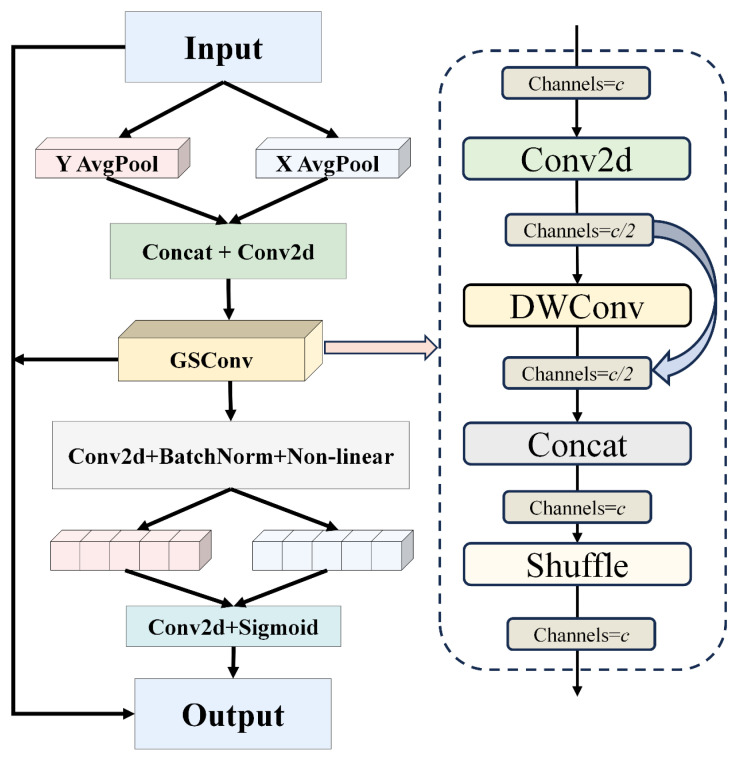
CAGS network structure.

To address these issues, this paper proposes integrating GSConv (Group Shuffle Convolution) [18] into the CA attention mechanism, forming the CAGS attention module. The network structure is depicted in Figure 3. Firstly, represent the input feature map as [C, H, W], where C denotes the number of channels, H denotes the height, and W denotes the width. Perform global average pooling separately along the width and height dimensions to obtain feature maps [C, 1, W] and [C, H, 1], respectively. Then, transpose these two feature maps to align them in the same dimensions, resulting in a merged feature map [C, H + W, 1]. Subsequently, process the merged feature map using GSConv operations.

The GSConv operation first applies standard convolution to the input feature map, resulting in a feature map with half the original number of channels, denoted as C/2. Subsequently, it applies Depthwise Separable Convolution (DWConv) [19] to reduce computational load by performing convolution operations independently across individual channels. After DWConv, another feature map with C/2 channels is obtained. These two feature maps are then concatenated to form a feature map with C channels. Finally, shuffle operations [20] are used to facilitate better feature fusion by promoting information interaction between different convolutional layers, resulting in the target number of output feature maps.

Process the feature maps obtained through GSConv operations with convolution, normalization, and non-linear activation functions. Then, split the feature maps into two parallel stages representing width and height information, resulting in two feature maps: [C, 1, H] and [C, 1, W]. Transpose these two feature maps to convert them into [C, H, 1] and [C, 1, W]. Use a 1 × 1 convolution layer to adjust the channel numbers and apply the sigmoid activation function to obtain attention weights in the width and height dimensions. Finally, fuse the resulting attention weights with the original input feature map and the feature map processed by CAGS to obtain the adjusted feature representation under the CAGS attention mechanism. This adjusted feature representation will be used for subsequent tasks in the model, enabling more effective capture of key information and enhancing overall performance.

#### 2.2.3. Optimized Loss Function

The loss function used in the original YOLOv7 model is the CIoU loss, with the formula as follows:

(1) $$CIoU=IoU-\frac{\rho^{2}\left(b,b^{gt}\right)}{c^{2}}-\alpha v$$

(2) $$\alpha =\frac{v}{1-IoU+v}$$

(3) $$v=\frac{4}{\pi^{2}}(\arctan\frac{w^{gt}}{h^{gt}}-\arctan\frac{w}{h}{)}^{2}$$

(4) $$IoU=\frac{A\cap B}{A\cup B}$$

where A is the predicted box and B is the ground truth box. $w^{gt}$ and $h^{gt}$ represent the height and width of the ground truth box, respectively, while w and h represent the height and width of the predicted box, respectively. The term v primarily measures the consistency of the aspect ratio, α is the balancing parameter, b and $b^{gt}$ represent the center points of the predicted and ground truth boxes, respectively, and ρ denotes the Euclidean distance between these two center points. c represents the diagonal length of the smallest enclosing box that can contain both the predicted and ground truth boxes.

Although CIoU loss considers three significant factors—aspect ratio, center point distance, and overlap area—when $w^{gt}$/$h^{gt}$ equals w/h, the term v becomes 0, as shown in Equation (3). This condition can lead to instability in the convergence of the CIoU loss function.

**Figure 4:**
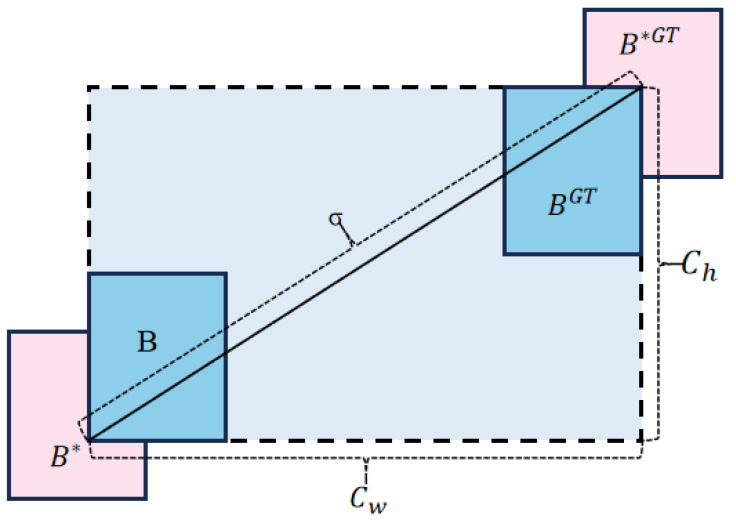
The scheme for calculation of angle cost and distance cost contribution into the loss function. B represents the predicted bounding box, $B^{gt}$ denotes the ground truth bounding box, and $B^{*}$ and $B^{*GT}$ are the minimum enclosing rectangles of the predicted and ground truth bounding boxes, respectively. ${C_{W}}^{\,}$ and ${C_{h}}^{\,}$ represent the width and height of the minimum enclosing rectangle, and $\alpha^{\,}$ indicates the angular difference between the bounding boxes.

To address this issue, this paper introduces SIoU to replace CIoU. SIoU primarily consists of three components: angle loss, distance loss, and shape loss, as illustrated in Figure 4.

Angle cost is defined as follows:

(5) $$\Lambda =1-2\times \sin^{2}\left(\arcsin\left(\frac{c_{h}}{\sigma }\right)-\frac{\pi }{4}\right)$$

where $c_{h}$ represents the height difference between the center points of the ground truth box $B^{*GT}$ and the predicted box $B^{*}$, and σ denotes the distance between their center points.

Distance cost is defined as follows:

(6) $$\Delta =\sum_{t=x,y}\left(1-e^{-\gamma \rho_{t}}\right)$$

(7) $$\rho_{x}=(\frac{b_{c_{x}}^{gt}-b_{c_{x}}}{c_{w}}{)}^{2},\rho_{y}=(\frac{b_{c_{y}}^{gt}-b_{c_{y}}}{c_{h}}{)}^{2},\gamma =2-\Lambda$$

where ($b_{c_{x}}^{gt},b_{c_{y}}^{gt}$) denotes the center coordinates of ground truth box $B^{GT}$, and ($b_{c_{x}},b_{c_{y}}$) denotes the center coordinates of predicted box B.

Shape cost is defined as follows:

(8) $$\Omega =\sum_{t=w,h}(1-e^{-w_{t}}{)}^{\theta }$$

(9) $$w_{w}=\frac{\left|w-w^{gt}\right|}{max(w,w^{gt})},w_{h}=\frac{\left|h-h^{gt}\right|}{max(h,h^{gt})}$$

where (w,h) and $\left(w^{gt},h^{gt}\right)$ represent the width and height of predicted box B and ground truth box $B^{GT}$ respectively. Parameter θ is used to control the emphasis on shape loss. To prevent excessive focus on shape loss and thereby reduce the movement of predicted boxes, θ is constrained to the range [3,7].

In conclusion, the final definition of the SIoU loss function is as follows:

(10) $${Loss}_{SIoU}=1-IoU+\frac{\Delta +\Omega }{2}$$

Compared to the CIoU loss function, SIoU incorporates angle cost, redefines the penalty metric, and avoids instability in model convergence when the aspect ratios of the ground truth and predicted boxes are equal.

## 3. Result and Discussion

### 3.1. Evaluating Indicator

This study adopts Precision (P), Recall (R), mAP@0.5, mAP@0.95, and single-image detection time as evaluation metrics. Precision (P) refers to the ratio of correctly predicted positive samples to all samples predicted as positive. Recall (R) refers to the ratio of correctly predicted positive samples to all actual positive samples, calculated as follows:

(11) $$P=\frac{TP}{TP+FP}\times 100\%$$

(12) $$R=\frac{TP}{TP+FN}\times 100\%$$

True Positive (TP) represents the number of samples that are actually positive and predicted as positive. False Positive (FP) refers to the number of samples that are actually negative but predicted as positive. False Negative (FN) represents the number of samples that are actually positive but predicted as negative.

The Precision–Recall (PR) curve is a common metric for assessing model performance, with Precision plotted on the vertical axis and Recall on the horizontal axis. Average Precision (AP) is a scalar representation of the area under the PR curve, with higher values indicating better classifier performance. Mean Average Precision (mAP) represents the average of AP across all detected classes.

(13) $$AP=\int_{0}^{1}P(R)dR$$

(14) $$mAP=\frac{\sum AP}{N(class)}$$

mAP@0.5 represents the mAP value at an IoU threshold of 0.5. mAP@0.95 represents the average mAP across all 10 IoU thresholds, ranging from 0.5 to 0.95 with a step size of 0.05.

The ROC curve (Receiver Operating Characteristic Curve) is a tool used to evaluate the performance of binary classification models, widely applied in machine learning and statistics. The ROC curve illustrates the relationship between the True Positive Rate (TPR) and the False Positive Rate (FPR) to describe the model’s classification ability. The closer the ROC curve is to the upper-left corner, the better the model’s performance. The AUC (Area Under the Curve) is a numerical measure of the ROC curve, and a value closer to 1 indicates better overall predictive performance.

(15) $$TPR=\frac{TP}{TP+FN}$$

(16) $$FPR=\frac{FP}{FP+TN}$$

AUC (Area Under the Curve) is a numerical metric that quantifies the performance of a classification model based on the ROC curve. It represents the area under the ROC curve and reflects the model’s ability to distinguish between positive and negative classes. The range of AUC values is from 0 to 1, and the higher the AUC value, the better the model performance.

(17) $$AUC=\frac{\sum \left(p_{i},n_{j}\right)}{P\times N}$$

where P is the number of positive samples and N is the number of negative samples. $p_{i}$ is the positive sample prediction score, which is the probability of predicting a positive sample as a positive example; $n_{j}$ is the negative sample prediction score, which is the probability of predicting a negative sample as a positive example. Use an indicator function to represent the positive and negative sample pairs in the above equation where the predicted positive sample value $p_{pos}$ is greater than the predicted negative sample value $p_{neg}$.

(18) $$AUC=\frac{\sum I(p_{pos},p_{neg})}{P\times N}$$

(19) $$I(p_{pos},p_{neg})=\left\{\begin{array}{c} 1\;\;\;\;\;\;p_{pos}>p_{neg}\\ 0.5\;\;\;\;p_{pos}=p_{neg}\\ 0\;\;\;\;\;\;\;p_{pos}<p_{neg} \end{array}\right.$$

### 3.2. Model Training

The CPU used in this study is an Intel (R) Xeon (R) Gold 6330 CPU @ 2.00 GHz, with 256 GB of memory and a 2 TB hard drive. The GPU used is an NVIDIA A100 with 80 GB of memory. The programming language employed was Python version 3.8, with PyTorch version 1.10, and CUDA version 11.4. The experimental parameter settings are shown in Table 1.

**Figure 5:**
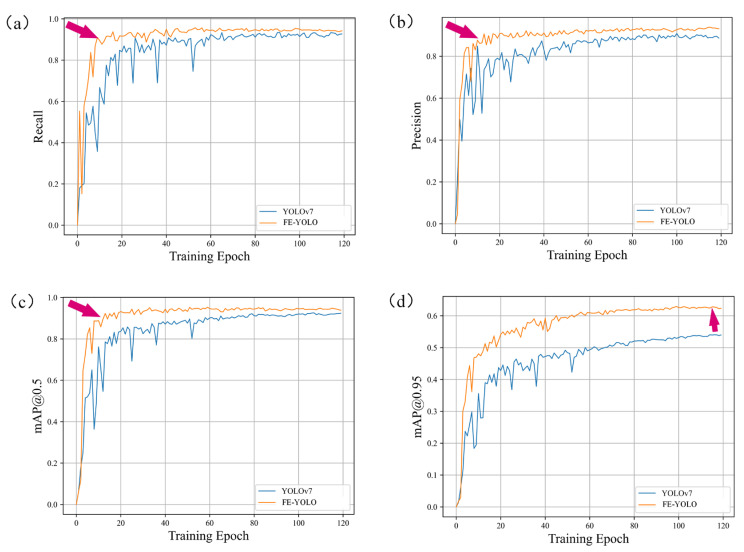
The progress of training performance of different models. (**a**) Recall, (**b**) Precision, (**c**) mAP@0.5, (**d**) mAP@0.95.

In our study, the dataset was then divided into training, validation, and testing sets at an 8:1:1 ratio. Subsequently, to evaluate the effectiveness of FE-YOLO compared to YOLOv7, we conducted experiments on the training set. During the training process, the curves depicting the changes in Recall, Precision, mAP@0.5, and mAP@0.95, along with comparisons to the original YOLOv7 model, are illustrated in Figure 5. Overall, FE-YOLO shows improvements across various metrics compared to YOLOv7. As shown in Figure 5a,b, the Recall and Precision of FE-YOLO converge around the tenth epoch, whereas those of YOLOv7 converge around the twentieth epoch. Moreover, FE-YOLO exhibits higher Recall and Precision compared to YOLOv7. From Figure 5c, it can be observed that FE-YOLO shows a similar convergence speed in mAP@0.5 compared to YOLOv7 but achieves a slightly higher value, improving by approximately 2%. Figure 5d demonstrates that the mAP@0.95 of the FE-YOLO shows a significant improvement over YOLOv7, with an increase of approximately 8%, indicating a substantial enhancement in detection accuracy. These experiments demonstrate that the proposed FE-YOLO model shows significant performance improvements compared to the original YOLOv7 model.

**Figure 6:**
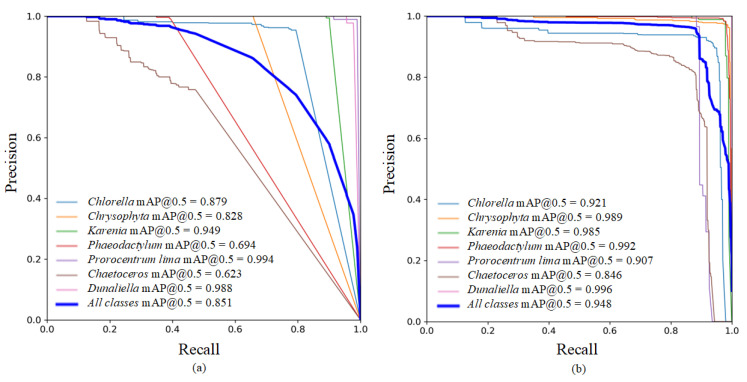
The comparison of P-R curves during training. (**a**) YOLOv7, (**b**) FE-YOLO.

The PR curves during the training process are shown in Figure 6, with the horizontal axis representing Recall and the vertical axis representing Precision. When the PR curve is closer to the top right corner, it indicates that the model can achieve both high Precision and high Recall in its predictions, implying more accurate results. In the figure, the seven differently colored curves represent the PR curves for seven different types of algae, while the thicker blue curve represents the average PR curve across all categories. From the figure, it is evident that the PR curves of the FE-YOLO are closer to the upper right corner, indicating that the FE-YOLO demonstrates superior performance.

**Figure 7:**
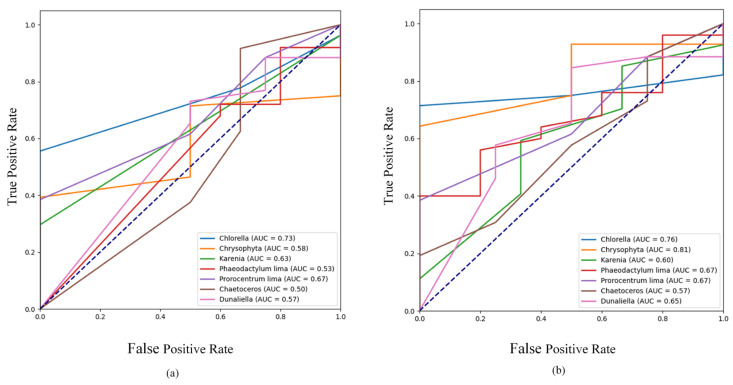
The comparison of ROC curves during training. (**a**) YOLOv7, (**b**) FE-YOLO. Dashed line: randomly guessing the baseline.

The ROC curve during the training process is presented in Figure 7, where the horizontal axis represents the False Positive Rate (FPR) and the vertical axis represents the True Positive Rate (TPR). A curve that is closer to the top-left corner indicates better predictive performance of the model [21]. In the figure, the seven differently colored curves correspond to the ROC curves for the seven types of algae. It is evident from the figure that the ROC curve for FE-YOLO is closer to the upper-left corner, signifying its superior performance. Additionally, the AUC (Area Under the Curve) values are calculated and displayed in the figure, where a higher AUC value, approaching 1, indicates better model performance. As shown, FE-YOLO consistently demonstrates superior performance compared to the other models.

### 3.3. Ablation Experiments

This paper introduces two improvements to the original YOLOv7 model. To further investigate the impact of different modules on recognition results, ablation experiments were conducted, with each experiment incorporating only one improvement method. The ablation experiment results for different microalgae are shown in Table 2.

The experimental results indicate that incorporating the CAGS module into the YOLOv7 model and replacing the CIoU loss function with SIoU both lead to significant improvements in detection accuracy, Recall, and mAP metrics. Based on the results of these ablation experiments, the FE-YOLO model proposed in this paper shows significant enhancements over the original YOLOv7 model: Precision increased from 85.2% to 94.8%, an improvement of 9.6%; Recall increased from 94.2% to 96.1%, an improvement of 1.9%; mAP@0.5 increased from 85.1% to 94.8%, an improvement of 9.7%; and mAP@0.95 increased from 62.4% to 69.3%, an improvement of 6.9%.

Furthermore, the detection time per single image decreased from 0.0501 s to 0.0455 s, indicating a speed improvement of 9.2%. Overall, under the same experimental conditions, FE-YOLO has shown significant improvements over the original YOLOv7 model in both detection accuracy and speed.

**Figure 8:**
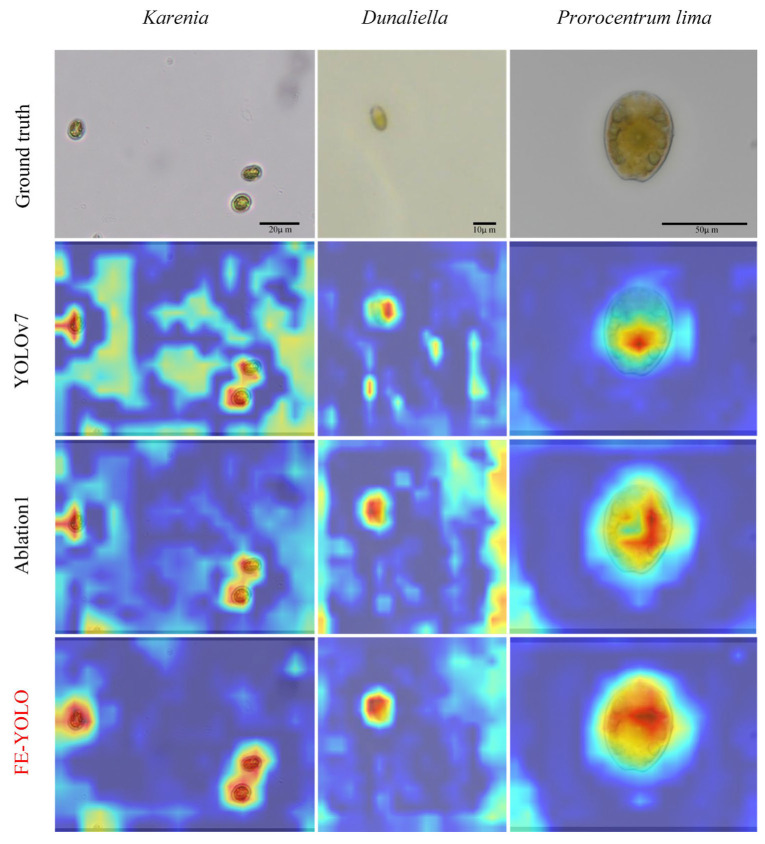
Visualization of attention maps for the YOLOv7, ablation1, and FE-YOLO on the microalgae dataset.

Figure 8 illustrates the complex challenges faced by the ground truth in identifying microalgae targets, especially in areas where the contours of the microalgae are indistinct or blurred. In these regions, it is difficult to distinguish the contour features of the microalgae from the background. This impedes the model’s ability to accurately capture and identify the targets, significantly affecting the recognition performance of the algorithm. Such limitations can lead to misjudgments in the detection process, thereby reducing the overall accuracy and reliability of detection. In contrast, our proposed improvements have optimized the algorithmic architecture, enabling the differentiation of microalgae from the background. This enhancement significantly improves the accuracy of localization and the Precision of microalgae detection.

### 3.4. Comparative Experiment

**Figure 9:**
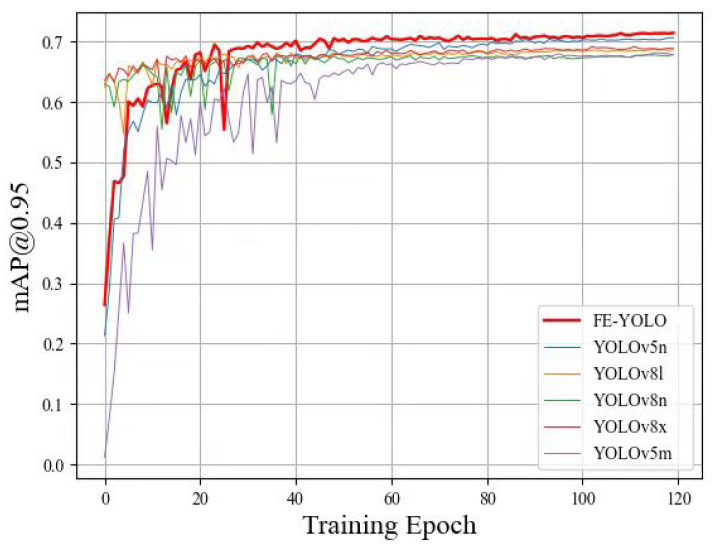
mAP@0.95 curves for different methods on the microalgae dataset.

To validate the performance of the proposed method, we compared the FE-YOLO algorithm with the latest classical algorithms, including Faster RCNN [22], YOLOv6 [23], DETR [24], YOLOv5 [25] and YOLOv8 [26]. Table 3 presents the detection results of all methods on the microalgae dataset, and Figure 9 displays the corresponding mAP@0.95 curves during the training process for a more intuitive comparison of the performance of various methods. The results indicate that the proposed FE-YOLO algorithm demonstrates significant improvements in Recall, mAP@0.5, and mAP@0.95 compared to benchmark methods. As seen in Table 3, FE-YOLO has a significant advantage in both detection Precision and Recall, indicating that it can obtain more accurate localization and higher quality prediction frames, mainly due to the more adequate feature fusion and robust semantic information of the CAGS module. Moreover, while maintaining high detection accuracy, the number of parameters and GFLOPS in our model remains small, which is mainly attributed to the two lightweight detection heads we propose. In summary, the FE-YOLO model proposed in this paper demonstrates superior performance in microalgae object detection compared to the latest mainstream object detection algorithms.

### 3.5. Microalgae Detection Results

**Figure 10:**
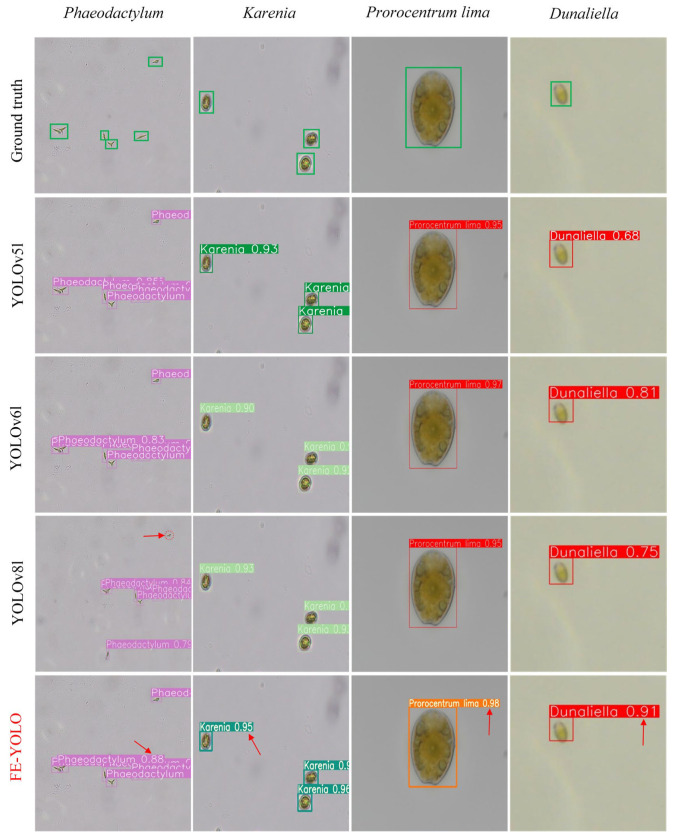
The detection results of different methods.

To visually validate the effectiveness of the proposed method, we compared the detection results of the FE-YOLO algorithm with the latest classical algorithms—YOLOv5l, YOLOv6l and YOLOv8l—on images of different microalgae species. Some of the detection results are shown in Figure 10. The experiment demonstrates that the three classic algorithms exhibit low detection accuracy and a high rate of missed detections. For instance, YOLOv8l fails to detect *Phaeodactylum*, while YOLOv5l and YOLOv6l show poor accuracy in detecting *Karenia*, *Prorocentrum lima*, and *Dunaliella*. These results highlight that, in comparison to classic algorithms such as YOLOv5l, YOLOv6l, and YOLOv8l, the FE-YOLO method achieves superior detection performance across different algae species.

## 4. Conclusions

Addressing the current challenges of slow detection speed and low accuracy in microalgae detection, this paper proposes an FE-YOLO algorithm based on the YOLOv7 model. To enhance detection accuracy, the CAGS module was integrated into the Neck section of YOLOv7. To address the issue of unstable convergence, SIoU was adopted as the loss function, resulting in further improvements in detection accuracy. To validate the effectiveness of the proposed algorithm, this study employed a dataset consisting of 6300 microscope images of seven common microalgae samples (*Chaetoceros*, *Chlorella*, *Chrysophyta*, *Prorocentrum lima*, *Karenia*, *Dunaliella*, and *Phaeodactylum*). The dataset was established through microscopy and compared against recent classical algorithms, including DETR, Faster RCNN, YOLOv5, YOLOv6, YOLOv7, and YOLOv8. The experimental results demonstrate that the FE-YOLO model shows significant improvements over the latest classical algorithms in terms of average Precision, Recall, mAP@0.5, and mAP@0.95.

## Figures and Tables

**Table 1:** Parameter settings during training.

Parameter	Configuration
Learning rate Momentum Weight decay Batch size Works Epochs Image size	0.01 0.937 0.0005 16 8 120 640 × 640

**Table 2:** Comparison results of ablation experiments on the microalgae dataset.

AP														
Method	CAGS	SIOU	Chl	Chr	Kar	Pha	Pro	Cha	Dun	P	R	mAP@0.5	mAP@0.95	Ds
YOLOv7	×	×	87.9%	82.8%	94.9%	69.4%	99.4%	62.3%	98.8%	85.2%	94.2%	85.1%	62.4%	0.0501 s
Ablation1	√	×	88.2%	83.6%	95.2%	73.5%	99.4%	66.7%	98.7%	86.4%	94.6%	88.9%	67.8%	0.0500 s
FE-YOLO	√	√	**92.1%**	**98.9%**	**98.5%**	**99.2%**	**90.7%**	**84.6%**	**99.6%**	**94.8%**	**96.1%**	**94.8%**	**69.3%**	**0.0455 s**

Chl: *Chlorella*, Chr: *Chrysophyta*, Kar: *Karenia*, Pha: *Phaeodactylum*, Pro: *Prorocentrum lima*, Cha: *Chaetoceros*, Dun: *Dunaliella*, Ds: Detection speed.

**Table 3:** Comparison results with other methods.

Method	R	mAP@0.5	mAP@0.95	GFLOPS	Parameters
Faster RCNN	85.0%	84.9%	41.6%	207	40 M
DETR	90.5%	92.2%	51.3%	225	41 M
YOLOv5l	94.7%	95.0%	65.0%	107.7	46.5 M
YOLOv5x	93.3%	93.9%	65.4%	203.9	86.7 M
YOLOv6m	94.0%	95.6%	64.8%	85.8	34.9 M
YOLOv6l	94.5%	94.9%	63.8%	150.7	59.6 M
YOLOv7	94.2%	93.2%	56.3%	103.2	36.5 M
YOLOv8n	72.7%	84.8%	62.4%	8.1	30.0 M
YOLOv8l	72.8%	84.7%	64.2%	164.8	43.6 M
FE-YOLO	**96.1%**	**94.8%**	**69.3%**	**98.7**	**26.3 M**

## Data Availability

The authors will supply the relevant data in response to reasonable requests.
